# Effectiveness of non-interruptive nudge interventions in electronic health records to improve the delivery of care in hospitals: a systematic review

**DOI:** 10.1093/jamia/ocad083

**Published:** 2023-05-15

**Authors:** Magdalena Z Raban, Peter J Gates, Sarah Gamboa, Gabriela Gonzalez, Johanna I Westbrook

**Affiliations:** Centre for Health Systems and Safety Research, Australian Institute of Health Innovation, Macquarie University, Sydney, Australia; Centre for Health Systems and Safety Research, Australian Institute of Health Innovation, Macquarie University, Sydney, Australia; National Drug and Alcohol Research Centre, University of New South Wales, Sydney, Australia; Centre for Health Systems and Safety Research, Australian Institute of Health Innovation, Macquarie University, Sydney, Australia; Centre for Health Systems and Safety Research, Australian Institute of Health Innovation, Macquarie University, Sydney, Australia; Centre for Health Systems and Safety Research, Australian Institute of Health Innovation, Macquarie University, Sydney, Australia

**Keywords:** economics, behavioral, medical records systems, computerized, medical informatics, quality of health care, systematic review

## Abstract

**Objectives:**

To describe the application of nudges within electronic health records (EHRs) and their effects on inpatient care delivery, and identify design features that support effective decision-making without the use of interruptive alerts.

**Materials and methods:**

We searched Medline, Embase, and PsychInfo (in January 2022) for randomized controlled trials, interrupted time-series and before–after studies reporting effects of nudge interventions embedded in hospital EHRs to improve care. Nudge interventions were identified at full-text review, using a pre-existing classification. Interventions using interruptive alerts were excluded. Risk of bias was assessed using the ROBINS-I tool (Risk of Bias in Non-randomized Studies of Interventions) for non-randomized studies or the Cochrane Effective Practice and Organization of Care Group methodology for randomized trials. Study results were summarized narratively.

**Results:**

We included 18 studies evaluating 24 EHR nudges. An improvement in care delivery was reported for 79.2% (*n* = 19; 95% CI, 59.5–90.8) of nudges. Nudges applied were from 5 of 9 possible nudge categories: change choice defaults (*n* = 9), make information visible (*n* = 6), change range or composition of options (*n* = 5), provide reminders (*n* = 2), and change option-related effort (*n* = 2). Only one study had a low risk of bias. Nudges targeted ordering of medications, laboratory tests, imaging, and appropriateness of care. Few studies evaluated long-term effects.

**Discussion:**

Nudges in EHRs can improve care delivery. Future work could explore a wider range of nudges and evaluate long-term effects.

**Conclusion:**

Nudges can be implemented in EHRs to improve care delivery within current system capabilities; however, as with all digital interventions, careful consideration of the sociotechnical system is crucial to enhance their effectiveness.

## INTRODUCTION

Despite the widescale adoption of electronic health records (EHRs) in hospitals to support the delivery of patient care, the impact of these systems on the use of evidence-based care and clinical outcomes is mixed.^1–5^ Clinical decision support (CDS) embedded in EHRs aims to support clinicians during their decision-making process. However, the design of effective clinical decision support (CDS) remains an ongoing challenge. A systematic review and meta-analysis of 108 studies found that CDS increased the percentage of patients receiving desired care by only 5.8% (95% CI, 4.0–7.6%).^1^ Furthermore, the effectiveness of the CDS within EHRs has not increased substantially over time. Safety performance testing of EHRs in the United States found that EHRs prevented 54.0% of potential adverse drug events tested in 2009; but in 2016, this only improved slightly to 61.6%.^6^

The over-reliance on interruptive alerts as the delivery mechanism for CDS can partly explain the lack of impact of CDS on care delivery. Performance testing of EHRs showed that alerts fired for 89% of medication orders that were considered clinically safe according to guidelines.^7^ High rates of clinically irrelevant alerts lead to “alert fatigue” with clinicians overriding alerts routinely without considering their clinical relevance.^8^ Studies have shown that between 46% and 100% of alerts are overridden, and that up to 100% of these overrides are appropriate.^9^ As a result, the effectiveness of clinically relevant alerts may be reduced. Furthermore, high rates of clinically irrelevant alerts contribute to clinician dissatisfaction with EHRs, and significant opportunity and productivity costs.^10^ Clearly, new approaches to the design of CDS are needed.

Nudge interventions,^11^ from the field of behavioral economics, provide a new approach to conceptualizing CDS design and can be applied without the use of interruptive alerts. Nudges are changes to the choice architecture within which decisions are made that encourage behavior change.^11^ In this case, the modules and views within an EHR represent a large number of choice architectures that support documentation and care decisions. For example, as the majority of people will remain with a default option, changing the default choice will shift the frequency with which that option is selected. The potential application of nudges in CDS design has been recognized,^12^^,^^13^ but not evaluated systematically. Three recent reviews on the use of nudge interventions in healthcare have examined nudges applied through various other modes, such as letters and education, but none have focused solely on nudges embedded in EHRs.^14–17^ Thus, we aimed to describe evidence of the types of nudges used in CDS within EHRs and their effects on care delivery, in order to inform future CDS design.

## METHODOLOGY

We conducted a systematic review of the literature following the Preferred Reporting Items for Systematic Reviews and Meta-Analyses (PRISMA) guidelines (Supplementary File S1) is the PRISMA checklist.^18^

### Information sources and search strategy

We searched 3 medical literature databases (Medline, Embase, and PsychInfo) for original articles published in English from 2009, the year the first edition of the book on nudges by Richard Thaler and Cass Sunstein was published.^11^ We used a combination of keywords and subject headings representing 3 key concepts: nudge interventions, EHRs, and hospitals. Reference lists of included articles were manually searched for potentially relevant studies. Finally, websites of ‘nudge’ units or organizations specializing in the application of nudge theory were also searched. Searches were conducted in January 2022. The full search strategy is shown in Supplementary File S2.

### Study selection process

Two researchers independently screened the title and abstracts of the literature searches, and then the full-text articles against the inclusion criteria. Quantitative evaluations of a nudge intervention embedded in the EHRs of a hospital with an aim to improve the delivery of care were included. The outcomes reported in studies were screened for those that measured the quality of care delivery, such as the appropriateness of medication use or test ordering. Studies examining the impact on improving documentation or patient billing were excluded. Eligible study designs were before–after studies, controlled before–after studies, interrupted time series, and randomized trials. Cross-sectional studies were excluded. Simulation studies or studies using simulated patients were excluded, as were studies in primary care, long-term care facilities and other non-hospital settings.

To be eligible, the intervention had to meet the definition of a nudge as proposed by Thaler and Sunstein: “A nudge… is any aspect of the choice architecture that alters people’s behaviour in a predictable way without forbidding any options or significantly changing their economic incentives”.^11^ In addition, we used the categories for nudge interventions proposed by Münscher to guide our decisions regarding whether an intervention was a nudge, and to categorize the nudges.^19^ We assessed whether interventions met the definition of a nudge at the full-text review stage, as abstracts often lacked sufficient detail to make this assessment solely based on the title and abstract. The nudge intervention had to be implemented within the EHR.

We excluded studies with interventions that used interruptive alerts since the aim of the review was to assess alternative design strategies to interruptive alerts. Studies implementing predictive algorithms or other machine learning approaches were also excluded as we were interested in parsimonious and pragmatic approaches to CDS design that EHR practitioners can implement readily within current system capabilities. Studies modifying existing order sets using nudges were included; however; we excluded studies implementing checklists and new order sets since the effectiveness of implementing these interventions has been examined comprehensively to date.^20–23^

### Data collection process and data items

Data were extracted by 2 researchers independently and verified by a third researcher. We extracted data on study characteristics, intervention description, and intervention effects. The interventions were classified according to the aforementioned taxonomy of choice architecture techniques, which consists of 3 broad categories: decision information, decision structure, and decision assistance.^19^Table 1 (from Münscher et al^19^) shows the techniques falling under each of the 3 categories with examples. Classification of the type of nudge intervention was done independently by 2 researchers, with discrepancies resolved through discussion.

**Table 1.** Classification of nudge interventions applied to included studies from Münscher et al^19^

Category	Technique	Technique examples
A. Decision information	A1. Translate information	Reframe information Simplify information
	A2. Make information visible	Provide real-time feedback Make external information visible
	A3. Provide social reference point	Refer to descriptive norm Refer to opinion leader
B. Decision structure	B1. Change choice defaults	Set no-action default Use prompted choice
	B2. Change option-related effort	Increase/decrease physical effort Increase/decrease financial effort
	B3. Change range or composition of options	Change categories of options Change grouping of options
	B4. Change option consequences	Connect decision to benefit or cost Change social consequences
C. Decision assistance	C1. Provide reminders	Make information more or less salient
	C2. Facilitate commitment	Support self-commitment/public commitment

### Risk of bias assessment

Study risk of bias was assessed using the ROBINS-I tool (Risk of Bias in Non-randomized Studies of Interventions)^24^ for non-randomized studies and the Cochrane Effective Practice and Organization of Care Group (EPOC) methodology for randomized trials.^25^ Two reviewers independently assessed risk of bias and differences in assessment were resolved through discussion. The ROBINS-I tool assesses 7 domains of bias and provides a judgment score for each of either “low”, “moderate”, “serious”, “critical” risk of bias or “no information” where there is a lack of information to assess the domain. The EPOC tool comprises 9 domains of bias, including those specific to randomization such as sequence generation and allocation concealment, and is scored using “low risk”, “high risk”, or “unclear risk” for each domain.

We attributed an overall risk of bias assessment to each study assessed using the ROBINS-I tool as described in the tool.^24^ For randomized trials assessed using the EPOC tool, we assigned an overall assessment as follows: “low” when all domains were rated “low risk”; “moderate” where there was a maximum of 4 “high risk” ratings; “serious” where there were more than 4 domains rated “high risk”; “critical” where all domains were “high risk”; and “no information” where there was no clear indication that the study was at serious or critical risk of bias *and* there was “unclear risk” for one or more domains. Studies assessed to be of an overall critical risk of bias were excluded from data synthesis. Supplementary File S3 has the detailed definitions for the overall risk of bias assignment for both tools.

### Data synthesis

The variety of outcomes and study design in the included studies precluded meta-analysis. We summarized the overall effect of nudges on outcomes by calculating a percentage of studies reporting an improvement in outcomes, with 95% confidence intervals (CI) calculated using the Wilson method. The direction of the effect on outcomes that was considered an improvement varied between interventions. For example, one intervention may have aimed to increase the prescribing of a medication, while another aimed to reduce the use of a diagnostic test. Thus, we categorized the effect of interventions using the terminology “improvement” if there was a favorable change in outcomes; or “no change” when the intervention did not change outcomes. We used a harvest plot to provide an overview of whether an intervention improved outcomes or there was no change. Our harvest plot comprised a column to represent each nudge evaluated, with the column height proportionate to the overall risk of bias for each study. Effects of classes of nudge interventions were summarized narratively.

## RESULTS

**Figure 1.**
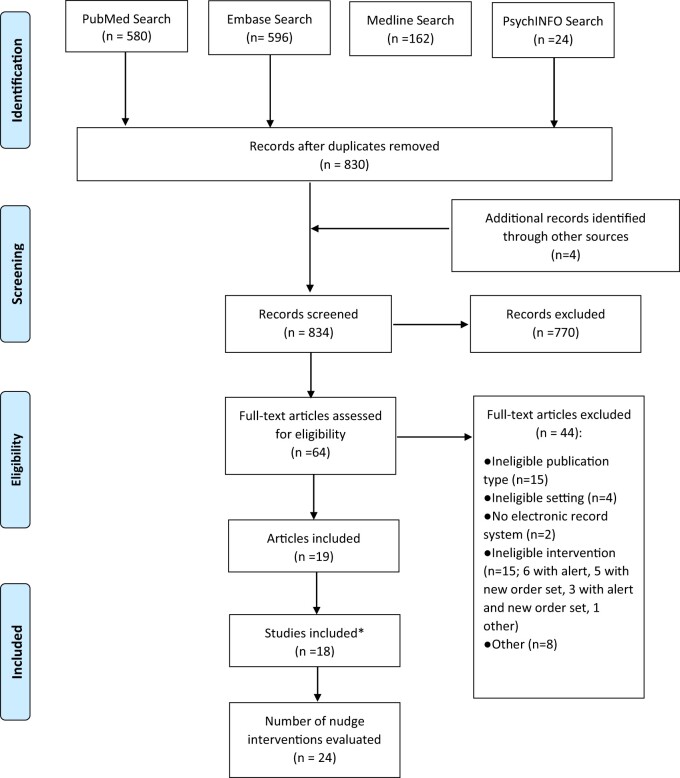
Preferred Reporting Items for Systematic Reviews and Meta-Analyses (PRISMA) diagram. *Two articles described one study.

From the 830 articles returned from the database searches and a further 4 articles from manual reference searching, we included 19 original research articles. Two articles reported on the same study^26^^,^^27^; with a final number of 18 included studies. Overall, the studies evaluated the impact of 24 nudge interventions within the EHR on care delivery (see PRISMA Flow Diagram in Figure 1).

Supplementary File S4 shows study characteristics. Fifteen studies were conducted in the United States,^26–41^ 2 in Canada,^42^^,^^43^ and 1 in England.^44^ Seven studies used a before–after design,^32–36^^,^^38^^,^^40^ 6 studies were interrupted time series,^28^^,^^29^^,^^37^^,^^41^^,^^42^^,^^44^ 4 were controlled before-after studies,^30^^,^^31^^,^^43^ and 1 was a randomized trial.^39^

Most studies were of moderate (*n* = 6) or serious risk of bias (*n* = 6), with only a single study found to be of low risk.^42^ Five studies did not have sufficient information in one or more domains to assess bias.^32^^,^^36–38^^,^^43^ The full risk of bias assessments for each study are provided in Supplementary Files S5 and S6.

### Nudge interventions implemented and their impact on care delivery outcomes

Five studies implemented multiple nudge interventions.^26^^,^^27^^,^^35^^,^^36^^,^^38^^,^^44^ Twelve interventions aimed to improve medication or fluid use^26–28^^,^^35^^,^^40–44^; 9 interventions to improve laboratory test ordering^29^^,^^31^^,^^32^^,^^34^^,^^36^^,^^38^^,^^39^; 2 interventions to improve appropriate care^33^^,^^37^; and 1 to improve imaging.^30^ Overall, studies used 5 of the 9 nudge interventions listed in the taxonomy applied: (1) change choice defaults (*n* = 9 interventions); (2) make information more visible (*n* = 6 interventions); (3) change range or composition of options (*n* = 5 interventions); (4) make information more or less salient (*n* = 2 interventions); and (5) change option-related effort (*n* = 2 interventions).

**Figure 2.**
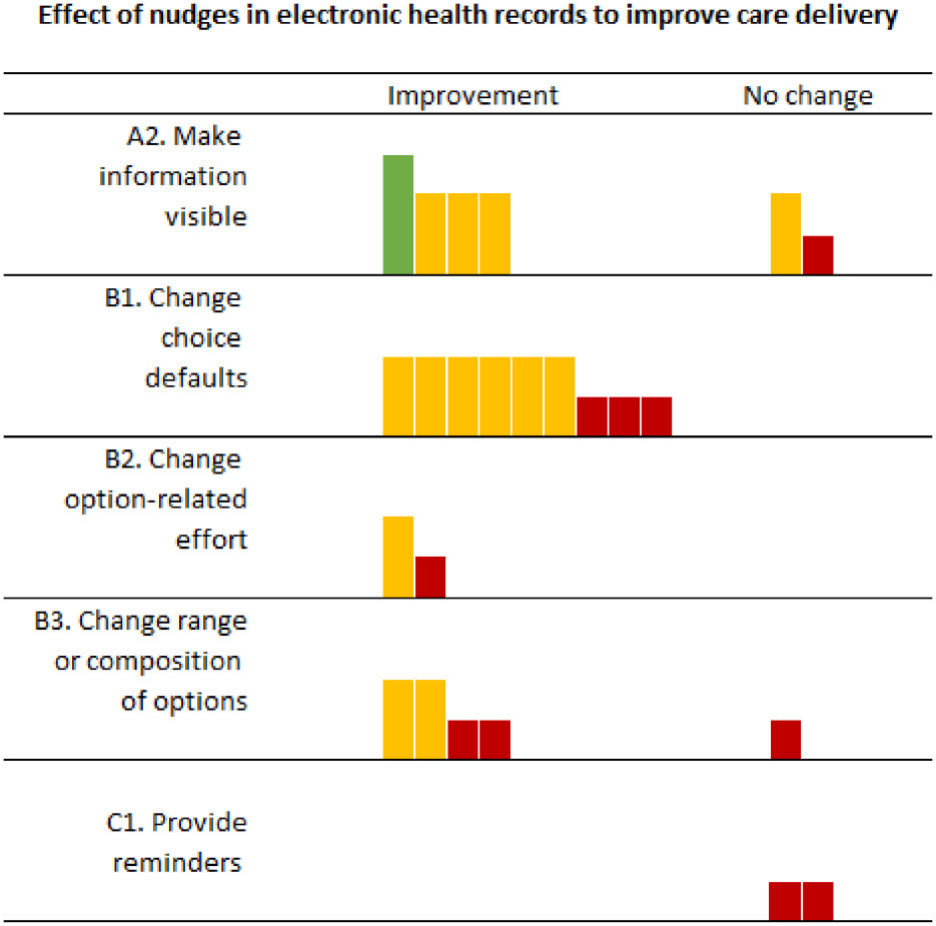
Harvest plot of effects of nudge interventions in electronic health records on care delivery outcomes. Each mark or column represents one nudge intervention. Column height and color represents the risk of bias in the study: tallest columns (green) are studies with low risk of bias; medium columns (yellow) are moderate risk of bias or not enough information; and short columns (red) are high risk of bias.

An improvement in care delivery was reported in 79.2% (*n* = 19; 95% CI, 59.5–90.8) of nudge interventions tested. Removing studies with a high risk of bias, the percentage of studies reporting an improvement in care delivery was 92.9% (*n* = 13; 95% CI, 68.5–98.7). Figure 2 is the harvest plot for the interventions included in the study and their impact on care delivery outcomes.

### Effects of “Make information more visible” nudge interventions

Six studies used the nudge of making information more visible in the EHR. Fived aimed to reduce inappropriate laboratory test orders^29–31^^,^^38^^,^^39^ and one inappropriate antibiotic ordering.^42^ Four of 6 interventions displayed the cost of test ordering,^30^^,^^31^^,^^38^^,^^39^ one changed the visibility of test options on an ordering quick list,^29^ and one made visible a pharmacist’s interpretation of a positive C difficile polymerase chain reaction (PCR) result.^42^ Details of the interventions implemented are shown in Table 2.

**Table 2.** Description of decision information nudge interventions in electronic health records

Nudge category/author, year	Intervention aim	Intervention description	Intervention effect
**Make information visible**			
Coughlin, 2020^29^	Reduce inappropriate urine analysis (UA)	Quick list order options for urine testing changed from: (1) urine analysis (UA) and (2) urine culture; to: (1) UA with reflex culture (urinary tract infection [UTI] symptoms present) and (2) UA w/reflex micro only (no UTI symptoms present)	Decrease of 2.98 urine cultures per 100 orders (95% CI, 1.45–4.51)
Durand, 2013^30^	Reduce imaging orders	Displayed costs of inpatient imaging at the point of ordering	No change in mean laboratory test ordering and the total charged costs of test orders
Herman, 2021^42^	Reduce inappropriate antibiotic use	Provision of additional pharmacist lead interpretation of a positive C difficile polymerase chain reaction (PCR) result	Decrease in the rate of antibiotic orders from 13.6 (10–16) to 7.9 days (1–13)—(difference −5.8 days; 95% confidence interval, −3.9 to −7.6); Increase in proportion of patients receiving no antibiotics from 6.5% to 23.6% (OR, 4.5; 95% CI, 2.3–8.7)
Feldman, 2013^31^	Reduce laboratory test ordering	Displayed costs of inpatient laboratory tests at point of ordering	Reduction in number of tests ordered by 9.1% (*P* < 0.001); Reduction in tests ordered/patient-day from 3.72 to 3.40 (8.59% decrease; 95% confidence interval: –8.99% to –8.19%); Net charge reduction of $436 115 (all *P* < 0.001)
Sadowski, 2017^38^	Reduce laboratory test ordering	Displayed costs of inpatient laboratory tests when a laboratory test was selected	15.3% (*P* < 0.00) reduction in number of tests ordered/patient day
Sedrak, 2017^39^	Reduce laboratory test ordering	Displayed costs of inpatient laboratory tests at point of ordering	No change in the number of tests ordered/patient day

Four of the 6 studies reported an improvement in outcomes^29^^,^^31^^,^^38^^,^^42^; 1 was rated at low risk of bias,^42^ 2 at moderate risk,^29^^,^^31^ and 1 did not provide enough information to assess risk of bias (Figure 2; Supplementary File S7 provides details of effects).^38^ Two studies reported no significant change in outcomes, both investigating the display of test cost information; one rated to have a serious risk of bias^30^ and one randomized controlled trial with a moderate risk of bias.^39^ Three studies collected data for 1 year,^29^^,^^39^^,^^42^ and 2 reported a sustained impact of the intervention across this time period.^29^^,^^42^ However, a randomized controlled trial reported a sustained lack of overall change, though explained this was likely due to differences in patient groups.^39^ That is, a significant decrease in test ordering reported among patients in intensive care was offset by a significant increase in test ordering for patients outside of intensive care. No studies examined intervention impact for longer than 1 year.

### Effects of “Change choice default” nudge interventions

Nine interventions used the nudge of changing default options to influence medication or fluid use, laboratory testing and appropriate care, and all studies reported an improvement in outcomes (Figure 2; Supplementary File S7 shows full results). Details of interventions implemented are shown in Table 3. Three studies were at serious risk of bias,^27^^,^^33^^,^^41^^,^^44^ 3 (reporting on a total of 4 interventions) at moderate risk,^26^^,^^28^^,^^36^ and 2 did not provide enough information to assess risk of bias.^37^^,^^43^ All studies reported substantial changes in ordering patterns following modifications to the default settings. In one study aiming to increase platelet count ordering post blood transfusion, the authors reversed this default to confirm patterns in ordering were indeed linked to the default setting. The orders for platelet counts were placed for 7%, 59.4%, and 7.5% of patients pre-intervention, post-intervention, and once the intervention was reversed, respectively.^36^ Three studies that collected data over one or more years reported sustained statistically significant impacts from this nudge intervention.^26^^,^^28^^,^^44^

**Table 3.** Details of decision structure nudge interventions in electronic health records

Nudge category/author, year	Intervention aim	Intervention description	Intervention effect
**Change choice defaults**			
Astroga, 2019^28^	Reduce antibiotic use	Automatic stop on antibiotic orders after 48 h coverage	Rate reduction of 25% (*P* < .0001) for parenteral antibiotic use/patient day
Bourdeaux, 2014^44^	Increase use of chlorhexidine mouthwash	Chlorhexidine mouthwash added to admission order set as an automatic order	Increase in percentage of prescriptions for mouthwash per patient per month from 55.3% to 90.4%
Delgado, 2018^41^	Reduce the number of opioid tablets prescribed at discharge	Changed default to 10 or 20 tablets on discharge prescription from no default	Increase in proportion of prescriptions for 10 tablets from 20.6% to 43.3% (difference of 22.8%, 95% CI, 19.6–25.9%); Decrease in proportion of prescriptions for 20 tablets from 22.8% to 16.1% (difference of –6.7%, 95% CI, –9.4% to –4%); No change in mean number of tablets prescribed (*P* = 0.42)
Jacobs, 2012^33^	Increase ordering of evidence-based care for asthma admissions	Order set default was set to include “weight”, “peak flow pre-post treatments” and “may go to activity centre” orders	Increase in percentage of patients with order for admission weight (79.2% to 94.8%, *P* < 0.001), activity center (84.1–95.3%, *P* < 0.001) and peak flow (18.8–55.9%, *P* < 0.001)
Leis, 2014^43^	Reduce antibiotic use for asymptomatic bacteriuria	Elimination of default reporting of urine culture results for non-catheterized patients	There was an absolute risk reduction of 36% (95% CI, 15%–57%) in asymptomatic bacteriuria treatment
Olson, 2015^36^	1. Increase hematocrit ordering post red cell transfusion 2. Increase platelet count orders post platelet transfusion	1. The posttransfusion order set had the default setting changed from “optional” to “preselected” for hematocrits 2. The default settings for platelet count was changed from “optional” to “preselected”	Increase from 8.3% to 57.5% (*P* < 0.0001) in percentage of transfusions with hematocrit count orders; Increase from 7.0% to 59.4% (*P* < 0.001) in percentage of transfusions with platelet count orders
Rubins, 2019^37^	Reduce unnecessary telemetry monitoring	Default in order set for telemetry monitoring was removed	Reduction in telemetry ordering from 79.1% of patients to 21.3% (*P* < 0.001)
Gerard, 2008^26^; Trick, 2009^^27^	Increase influenza vaccination	Introduced preselected vaccination orders by default triggered by a patient discharge order	Increase in percentage of patients vaccinated for influenza to 36% (compared to <5% for controls in previous year, *P* < 0.001)^
**Change range or composition of options**			
Muniga, 2020^35^	1. Increase ordering of scheduled acetaminophen 2. Increase H2RA ordering	1. Scheduled acetaminophen was added to the order set (only as needed acetaminophen appeared in the order set previously) 2. An H2RA was added to the order set (only a PPI appeared in the order set previously)	No change in percentage of patients with orders for scheduled acetaminophen; Increase in percentage of patients prescribed an H2RA for stress ulcer prophylaxis (0 vs 20%, *P* < 0.001)
Munigala, 2018^34^	Reduce urine culture ordering	Removed orders for “urinalysis with reflex to culture”, “urine macroscopic” and “urine microscopic” from the “frequently ordered tests” window and retained only “urinalysis with reflex to microscopy”	Decrease in daily culture rate per 100 patients by 46.6% (95% CI, −66.2% to –15.6%)
Sadowski, 2017^38^	Reduce laboratory test ordering	Daily laboratory test order removed from order set	Decrease in number of routine tests ordered/patient day from 4.99 to 4.02 (*P* <.001)
Smith, 2019^40^	Reduce opioid use	Combination opioids were eliminated from the post-caesarean order set and replaced with nonopioids	Reduction of 75% in morphine milligram equivalents/hospital stay (from 120 [90–176 IQR] to 30 [5–68] post-intervention [*P*<.001])
**Change option-related effort**			
Iturrate, 2016^32^	Reduce laboratory test ordering	Eliminated the order for recurring daily laboratory tests. Had to order daily tests manually	Reduction of 8.52% in number of laboratory tests ordered (*P* < 0.001); Estimated savings of $323 489
Bourdeaux, 2014^44^	Reduce use of HES	HES was removed from admission order set	Decrease in percentage of patients receiving HES/month from 54.1% to 3.1% (*P* < 0.001)

HES: hydroxyethyl starch infusion; H2RA: histamine-2 receptor antagonist; PPI: proton pump inhibitor.

^This result is for two interventions implemented simultaneously, i.e. pre-selected vaccine order for doctors and reminder for nurses.

### Effects of “Change option-related effort” nudge interventions

Two studies examined the impact of nudges in the category changing option-related effort with both reporting an improvement in outcome (Figure 2). A description of the interventions is presented in Table 3. One study, rated to be of serious risk of bias, assessed the impact of removing hydroxyethyl starch from a default order set.^44^ A significant reduction in the percentage of patients receiving hydroxyethyl starch per month was reported and maintained for 42 months. A second study, that did not provide enough information to assess risk of bias, evaluated the impact of eliminating a recurring order for daily laboratory tests.^32^ A significant reduction in laboratory test ordering was found; however, this outcome returned to baseline rates within 1 year of intervention implementation.

### Effects of “Change range or composition of options” nudge interventions

Four studies assessed the impact of 5 interventions that added^34^^,^^35^ or removed medication orders or laboratory tests^38^^,^^40^ from existing EHR displays. In 3 studies, this was done in existing orders sets,^35^^,^^38^^,^^40^ while one study changed the options in the ‘frequently ordered tests’ window (Table 3 presents details of interventions).^34^ Four of 5 interventions showed an improvement on one or more of the measured outcomes (Figure 2).^34^^,^^35^^,^^38^^,^^40^ Two studies were rated to be at serious risk of bias,^34^^,^^35^ one at moderate risk,^40^ and one did not provide enough information to assess risk of bias.^38^ One intervention involving the addition of scheduled paracetamol (acetaminophen) to the orders set, showed no change in the percentage of patients with these orders from a baseline of 0%.^35^ No study assessed outcomes for more than 4 months.

### Effects of “Provide reminders” nudge interventions

Two studies (reported in 3 articles) evaluated the impact of providing reminders, specifically by increasing the saliency of options, and neither reported an improvement in outcomes (Figure 2).^26^^,^^27^^,^^35^ Both studies were assessed as having a serious risk of bias.^26^^,^^27^^,^^35^ Details of interventions are provided in Table 4. The first study assessed the impact of an automated reminder to vaccinate patients for influenza placed on the nurse activity list. No significant change in vaccination rates was observed over 3-month data collection periods with this intervention alone.^26^^,^^27^ The second study assessed the impact of listing lactated Ringer’s solution first in the fluid options on a selection list, placed above normal saline. That study reported an unexpected reduction in the use of lactated Ringer’s from 17% to 4% of patients at 3 months post-intervention (*P* = 0.005) and no significant change in the use of normal saline.^35^ The study authors considered this finding to reflect changes in the patient cohorts pre- and post-intervention, with the post-intervention group not requiring as many maintenance fluids as the pre-intervention group. Furthermore, they acknowledged that ordering of IV fluids often occurred in the emergency department which was out of the study scope and the providers in the study may have chosen to continue these orders once patients were on the study ward.

**Table 4.** Details of decision assistance nudge interventions in electronic health records

Nudge category/author, year	Intervention aim	Intervention description	Intervention effect
**Provide reminders (make information more or less salient)**			
Muniga, 2020^35^	Increase lactated Ringer’s solution use	Lactated Ringer’s solution was made the first fluid option on the selection list, above normal saline (normal saline was first option previously)	No change in the percentage of patients with an order for lactated Ringer’s compared to those with normal saline orders.
Gerard, 2008^26^; Trick, 2009^^27^	Increase influenza vaccination	Automatically added a reminder for vaccination to nurse activity list	No change compared to controls.

^This result is for two interventions implemented simultaneously, i.e. pre-selected vaccine order for doctors and reminder for nurses.

## DISCUSSION

In this systematic review, we report on the use of nudges embedded in EHRs to improve inpatient care. Five (of 9) categories of nudge interventions were evaluated across 18 studies: (1) changing choice defaults; (2) making information more visible; (3) changing the range or composition of options; (4) making information more or less salient; and (5) changing option-related effort. While the capacity for CDS to improve patient care has been established for some time,^45^ the results of improvements to CDS have often been underwhelming, particularly when interruptive alerts are used as the main mechanism for CDS delivery.^1^ Our findings show that 79.2% (*n* = 19; 95% CI, 59.5–90.8) of nudge interventions were successful in achieving improvements in care quality, without the use of interruptive alerts. However, all but one study was rated to have a moderate or serious risk of bias. In addition, long-term assessment of intervention impacts was limited.

Previous reviews of the use of nudge interventions in healthcare, but not specifically in EHRs, have highlighted that use of default options and the manipulation of choice salience are the most frequently evaluated nudges, and as a whole produce successful results.^14^^,^^46^ Our results are consistent with these reviews in that changing default choices was the most frequently evaluated and most consistently found to be effective. However, manipulating choice saliency was only assessed in 2 studies in our review and neither study reported the intervention to be effective.^26^^,^^27^^,^^35^

As supported in our results, the use of defaults in healthcare is a relatively cheap, easy, and a potentially very effective means to improve patient care^47^^,^^48^ and CDS system design. In principle, the effectiveness of default nudges lies in the “effort tax” required to reject the default and the degree to which the choosers believe that those designing the default options did so with good reason.^48^ As such, an important focus is to ensure this potential is used with care, particularly within CDS where system changes can bring about unintended consequences such as technology-related errors.^49^ That is, default options can guide a clinician toward appropriate care, but their use in EHRs places the onus of the decision of what is the most appropriate care on the system designer.

Recent reviews on the use of displaying price information, within or outside of CDS, indicate that this type of nudge intervention has promise in reducing test and medication ordering when the required resources to integrate the cost information are available.^50^^,^^51^ We identified 4 studies to assess this type of nudge intervention in CDS ordering choice,^30^^,^^31^^,^^38^^,^^39^ with 2 reporting a change in clinician ordering behaviors.^31^^,^^38^ The articles which showed no impact discussed the widely held belief that most imaging tests are expensive, so cost information may be generally ignored,^30^ and that cost information is considered to be of limited relevance as tests are generally deemed clinically appropriate.^39^ This highlights the need to carefully consider the context in which nudges are being implemented, and that a successful nudge in one setting may not be an effective strategy in another setting or when applied to a different aim.^52^

Changing defaults and making information visible were the most frequently tested nudges, with alternate types of nudge interventions less frequently assessed. However, we found that these alternate nudges still showed promise. For example, we identified 4 studies that assessed the impact of adding^34^^,^^35^ or removing medications or laboratory tests^38^^,^^40^ to existing order sets or shortcut lists in the EHR. Notably, all 4 studies reported a significant intervention impact on at least some of the measured outcomes. The effectiveness of order set implementation in EHRs on improved care quality, safety, and patient outcomes has been reported in the literature.^23^ Our results demonstrate that alterations to orders sets can also lead to improved outcomes. Further work is needed to ascertain the effectiveness of a broader range of nudges, aside from changing defaults and making information visible, and system designers should keep the full range of nudge strategies in mind when considering design options.

Evidence of the longevity of intervention impact was assessed in a limited number of studies. Three nudge intervention types were investigated for at least 1 year—change of choice default^26^^,^^28^^,^^44^; change of effort^32^^,^^44^; and changing choice saliency.^39^ All nudges that changed defaults reported that their effect was maintained over the study period. However, other nudges reported mixed effects over time, for example, the effects of changing choice effort returned to baseline over the study period in one study but were maintained in another, highlighting the need for long-term studies of the effectiveness of nudges.

Not all interventions were successful in improving care outcomes and some studies reported that they modified their intervention over the study period to facilitate better integration of the CDS into clinician workflows. Furthermore, one study reversed an intervention as it was deemed that it had shifted the test ordering to a magnitude that was likely inappropriate. These issues highlight the need for system designers to consider the complexity of the sociotechnical system within which they are implementing CDS, and the need for robust evaluation of the effects of any changes.^52^

Though we excluded studies implementing checklists and standardized order sets in this review, it is useful to examine the evidence for these interventions. Checklists and standardized order sets are a nudge that can be implemented on paper or in EHRs. Systematic reviews of both types of interventions report that they can be successful in improving adherence to care standards and patient outcomes.^21–23^ However, checklists and order sets are not without their limitations.^53^^,^^54^ Despite early successes of checklists in surgical care and the development of a World Health Organization Surgical Safety Checklist, evidence of early successes has been tempered by conflicting evidence on checklist effectiveness as implementation has been scaled.^53^^,^^54^ Due to emerging evidence of barriers to checklist uptake, adherence and hence effectiveness, checklists have been described as a “deceptively simple intervention”.^55^ Similarly, it is recognized that order set implementation has associated complexities, requiring clinicians to change their workflow to accommodate a new process.^21^^,^^23^ While all CDS design should be informed by an understanding of clinician workflow and the sociotechnical system more broadly, the nudges included in this review are relatively simple compared to checklists and order sets, in that they did not require clinicians to change their work processes. We believe that this is a strength of the CDS nudges used in the included studies.

As with all reviews, this review was limited by the quality of included studies. Our findings echo previous calls for further high-quality research on the use of nudges in healthcare.^14–16^ In addition, the design of studies was mostly limited to short term follow-up with only 5 studies collecting data for a minimum of 1 year.^26^^,^^28^^,^^32^^,^^39^^,^^42^^,^^44^ The variation in study outcomes and designs precluded a meta-analysis, limiting our reporting of results narratively. Nonetheless, the included studies provide examples of how nudges can be embedded in EHRs to improve care delivery.

## CONCLUSIONS

Effective CDS within EHRs requires careful design, and the overuse of interruptive alerts can compromise the effectiveness of CDS. Our review provides an overview of how nudge interventions can be implemented within EHRs to improve care delivery without the use of interruptive alerts. Changing choice defaults was the most frequently applied nudge in EHRs and delivered improvements in care delivery in all studies. However, other nudge strategies, while less frequently applied in EHRs, should also be considered in CDS design. While further evidence is needed on nudges in EHRs, including the sustainability of their effects, EHR managers and designers can use nudge interventions to inform CDS design while considering the local context. EHR vendors could examine ways to support the application of nudges in their systems, for example by including guidance about the design of order sentences and order sets, and use of defaults as potential elements in CDS design. Ideally, modifications to CDS should be monitored and evaluated to determine whether they are having the desired effect.

## Supplementary Material

ocad083_Supplementary_Data

## Data Availability

All data are available in the manuscript and supplementary files.
