# Supplementary material for: Effectiveness of non-interruptive nudge interventions in electronic health records to improve the delivery of care in hospitals: a systematic review
Source: J Am Med Inform Assoc. 2023 May 15;30(7):1313–22. doi: 10.1093/jamia/ocad083 (PMC10280359; doi:10.1093/jamia/ocad083)
Supplement: ocad083_Supplementary_Data [file ocad083_supplementary_data.pdf]

Supplementary files

Table of Contents

Supplementary File 1: Preferred Reporting Items for Systematic Reviews and Meta-Analyses (PRISMA) guidelines 2

Supplementary File 2: Search strategy 5

Supplementary File 3: Assignment of overall risk of bias score 9

Supplementary File 4: Characteristics of included studies examining effect of nudge interventions in electronic health records 10

Supplementary File 5: Quality assessment for non-randomised studies using the ROBINS-I tool (Risk of Bias in Non-randomised Studies of Intervention) 14

Supplementary File 6: Quality assessment of randomised trials using the Cochrane Effective Practice and Organisation of Care Group (EPOC) methodology 16

Supplementary File 7: Outcomes of included studies of nudge interventions in electronic health records and risk of bias 17

Supplementary File 1: Preferred Reporting Items for Systematic Reviews and Meta-Analyses (PRISMA) guidelines

Section and Topic	Item #	Checklist item	Location where item is reported
**TITLE**			
Title	1	Identify the report as a systematic review.	Page 1
**ABSTRACT**			
Abstract	2	See the PRISMA 2020 for Abstracts checklist.	Page 2
**INTRODUCTION**			
Rationale	3	Describe the rationale for the review in the context of existing knowledge.	Page 3
Objectives	4	Provide an explicit statement of the objective(s) or question(s) the review addresses.	Pages 3-4
**METHODS**			
Eligibility criteria	5	Specify the inclusion and exclusion criteria for the review and how studies were grouped for the syntheses.	Page 5-6
Information sources	6	Specify all databases, registers, websites, organisations, reference lists and other sources searched or consulted to identify studies. Specify the date when each source was last searched or consulted.	Page 5
Search strategy	7	Present the full search strategies for all databases, registers and websites, including any filters and limits used.	Supplementary file 2
Selection process	8	Specify the methods used to decide whether a study met the inclusion criteria of the review, including how many reviewers screened each record and each report retrieved, whether they worked independently, and if applicable, details of automation tools used in the process.	Pages 5-6
Data collection process	9	Specify the methods used to collect data from reports, including how many reviewers collected data from each report, whether they worked independently, any processes for obtaining or confirming data from study investigators, and if applicable, details of automation tools used in the process.	Page 6
Data items	10a	List and define all outcomes for which data were sought. Specify whether all results that were compatible with each outcome domain in each study were sought (e.g. for all measures, time points, analyses), and if not, the methods used to decide which results to collect.	Page 5
	10b	List and define all other variables for which data were sought (e.g. participant and intervention characteristics, funding sources). Describe any assumptions made about any missing or unclear information.	Pages 6, 8
Study risk of bias assessment	11	Specify the methods used to assess risk of bias in the included studies, including details of the tool(s) used, how many reviewers assessed each study and whether they worked independently, and if applicable, details of automation tools used in the process.	Page 7
Effect measures	12	Specify for each outcome the effect measure(s) (e.g. risk ratio, mean difference) used in the synthesis or presentation of results.	Page 8
Synthesis methods	13a	Describe the processes used to decide which studies were eligible for each synthesis (e.g. tabulating the study intervention characteristics and comparing against the planned groups for each synthesis (item #5)).	Page 8
	13b	Describe any methods required to prepare the data for presentation or synthesis, such as handling of missing summary statistics, or data conversions.	Page 8
	13c	Describe any methods used to tabulate or visually display results of individual studies and syntheses.	Page 8
	13d	Describe any methods used to synthesize results and provide a rationale for the choice(s). If meta-analysis was performed, describe the model(s),	Page 8

Section and Topic	Item #	Checklist item	Location where item is reported
		method(s) to identify the presence and extent of statistical heterogeneity, and software package(s) used.	
	13e	Describe any methods used to explore possible causes of heterogeneity among study results (e.g. subgroup analysis, meta-regression).	n/a
	13f	Describe any sensitivity analyses conducted to assess robustness of the synthesized results.	Page 8
Reporting bias assessment	14	Describe any methods used to assess risk of bias due to missing results in a synthesis (arising from reporting biases).	n/a
Certainty assessment	15	Describe any methods used to assess certainty (or confidence) in the body of evidence for an outcome.	n/a
**RESULTS**			
Study selection	16a	Describe the results of the search and selection process, from the number of records identified in the search to the number of studies included in the review, ideally using a flow diagram.	Page 9, figure 1
	16b	Cite studies that might appear to meet the inclusion criteria, but which were excluded, and explain why they were excluded.	n/a
Study characteristics	17	Cite each included study and present its characteristics.	Page 9, supplementary file 4
Risk of bias in studies	18	Present assessments of risk of bias for each included study.	Page 9, supplementary files 4, 5 and 6
Results of individual studies	19	For all outcomes, present, for each study: (a) summary statistics for each group (where appropriate) and (b) an effect estimate and its precision (e.g. confidence/credible interval), ideally using structured tables or plots.	Pages 9-15; figure 2; supplementary file 17
Results of syntheses	20a	For each synthesis, briefly summarise the characteristics and risk of bias among contributing studies.	Pages 9-15
	20b	Present results of all statistical syntheses conducted. If meta-analysis was done, present for each the summary estimate and its precision (e.g. confidence/credible interval) and measures of statistical heterogeneity. If comparing groups, describe the direction of the effect.	n/a
	20c	Present results of all investigations of possible causes of heterogeneity among study results.	n/a
	20d	Present results of all sensitivity analyses conducted to assess the robustness of the synthesized results.	Page 9
Reporting biases	21	Present assessments of risk of bias due to missing results (arising from reporting biases) for each synthesis assessed.	n/a
Certainty of evidence	22	Present assessments of certainty (or confidence) in the body of evidence for each outcome assessed.	n/a
**DISCUSSION**			
Discussion	23a	Provide a general interpretation of the results in the context of other evidence.	Page 16-17
	23b	Discuss any limitations of the evidence included in the review.	Page 18

Section and Topic	Item #	Checklist item	Location where item is reported
	23c	Discuss any limitations of the review processes used.	Page 18
	23d	Discuss implications of the results for practice, policy, and future research.	Page 18
**OTHER INFORMATION**			
Registration and protocol	24a	Provide registration information for the review, including register name and registration number, or state that the review was not registered.	n/a
	24b	Indicate where the review protocol can be accessed, or state that a protocol was not prepared.	n/a
	24c	Describe and explain any amendments to information provided at registration or in the protocol.	n/a
Support	25	Describe sources of financial or non-financial support for the review, and the role of the funders or sponsors in the review.	Page 19
Competing interests	26	Declare any competing interests of review authors.	Page 19
Availability of data, code and other materials	27	Report which of the following are publicly available and where they can be found: template data collection forms; data extracted from included studies; data used for all analyses; analytic code; any other materials used in the review.	Page 19

Supplementary File 2: Search strategy

Medline

1. *Choice Behavior/
2. *decision making/
3. *Economics, Behavioral/
4. Nudg*.tw.
5. "public commitment".tw.
6. "social norm feedback".tw.
7. "active choice".tw.
8. Choice Architecture*.tw.
9. opt in*.tw.
10. opt out*.tw.
11. opt out*.tw.
12. "human behaviour".tw.
13. 1 or 2 or 3 or 4 or 5 or 6 or 7 or 8 or 9 or 10 or 11 or 12
14. exp Medication Therapy Management/
15. *Electronic Prescribing/
16. *Medication Systems/
17. *medical records systems, computerized/ or *medical order entry systems/
18. *Diagnosis, Computer-Assisted/
19. *information systems/ or *decision support systems, clinical/ or *health information systems/ or *reminder systems/
20. *Data Collection/
22. *Medical Informatics/
23. "Electronic Medication Management".tw.
24. "eMM*".tw.
25. "Computeri*ed Physician Order Entry".tw.
26. "Computeri*ed Provider Order Entry".tw.
27. "CPOE".tw.
28. "EMR".tw.
29. e-Prescribing*.tw.
30. "health information technology".tw.
31. *Electronic Health Records/
32. 14 or 15 or 16 or 17 or 18 or 19 or 20 or 21 or 22 or 23 or 24 or 25 or 26 or 27 or 28 or 29 or 30 or 31
33. 13 and 32
34. limit 33 to yr="2008 -Current"
35. "hospital".tw.
36. ICU*.tw.
37. acute care*.tw.
38. "emergency department".tw.
39. "inpatient".tw.
40. hospitali*ation.tw.
41. 35 or 36 or 37 or 38 or 39 or 40
42. 13 and 32 and 41
43. limit 42 to yr="2008 -Current"inpatient*.tw.

Embase

1. *decision making/
2. behavioral economics/
3. Nudg*.tw.
4. public commitment*.tw.
5. social norm feedback*.tw.
6. active choice*.tw.
7. Choice Architecture*.tw.
8. opt in*.tw.
9. opt out*.tw.
10. human behaviour*.tw.
11. exp medication therapy management/
12. exp electronic prescribing/
13. exp computer assisted diagnosis/
14. information system/
15. information processing/
16. *medication therapy management/
17. "eMM*".tw.
18. "Computeri*ed Physician Order Entry".tw.
19. "Computeri*ed Provider Order Entry".tw.
20. *Medical Records Systems, Computerized/
21. "decision support system".tw.
22. "CPOE".tw.
23. "EMR".tw.
24. e-Prescribing*.tw.
25. *electronic health record/
26. "health information technology".tw.
27. *Medical Informatics/
28. exp physician order entry system/
29. *reminder system/
30. 1 or 2 or 3 or 4 or 5 or 6 or 7 or 8 or 9 or 10
31. 11 or 12 or 13 or 14 or 15 or 16 or 17 or 18 or 19 or 20 or 21 or 22 or 23 or 24 or 25 or 26 or 27 or 28 or 29
32. "hospital".tw.
33. ICU*.tw.
34. acute care*.tw.
35. "emergency department".tw.
36. "inpatient".tw.
37. hospitali*ation.tw.
38. 32 or 33 or 34 or 35 or 36 or 37
39. 30 and 31 and 38
40. limit 39 to yr="2008 -Current"

PsychINFO

1. *choice behavior/ or *behavior/ or *decision making/
2. *behavioral economics/
3. nudg*.tw.
4. nudg*.ti,ab.
5. public commitment*.tw.
6. social norm feedback*.tw.
7. active choice*.tw.
8. opt in*.tw.
9. opt out*.tw.
10. human behavio*.tw.
11. 1 or 2 or 3 or 5 or 6 or 7 or 8 or 9 or 10
12. exp medical records/ or exp health information technology/ or exp client records/ or exp electronic health records/
13. *decision support systems/ or *computer software/
14. medical order entry systems*.tw.
15. "medical order entry systems".tw.
16. *computer assisted diagnosis/
17. *information systems/
18. reminder systems*.tw.
19. *data collection/
20. Medical Informatics*.tw.
21. Electronic Medication Management*.tw.
22. eMM*.tw.
23. Computeri*ed Physician Order Entry*.tw.
24. Computeri*ed Provider Order Entry*.tw.
25. CPOE*.tw.
26. EMR*.tw.
27. e-Prescribing*.tw.
28. health information technology*.tw.
29. 12 or 13 or 14 or 15 or 16 or 17 or 18 or 19 or 20 or 21 or 22 or 23 or 24 or 25 or 26 or 27 or 28
30. ICU*.tw.
31. acute care*.tw.
32. emergency department*.tw.
33. inpatient*.tw.
34. hospitali*ation.tw.
35. 11 and 29
36. 30 or 31 or 32 or 33 or 34
37. 11 and 29 and 36
38. 11 and 29

PubMed

eMM Terms
((((((((((((((((((((((((Electronic Health Record*[MeSH Terms]) OR (Computerized Medical Record*[MeSH Terms]))) OR (Medical Record, Electronic[MeSH Terms])) OR ("health information technology"[Title/Abstract])) OR (Medical Records System*[MeSH Terms])) OR ("computer assisted diagnosis"[Title/Abstract])) OR (“information system”*Title/Abstract])) OR (“information processing”[Title/Abstract])) OR (electronic medication management[MeSH Terms])) OR (medication therapy management[MeSH Terms])) OR (computerized providers order entry[MeSH Terms])) OR (Medication Alert System*[MeSH Terms])) OR (Computerized Physician Order Entry System*[MeSH Terms])) OR ("decision support system*"[Title/Abstract])) OR ("EHR"[Title/Abstract])) OR ("CPOE"[Title/Abstract])) OR (“EMR”[Title/Abstract])) OR (“e-Prescribing”[Title/Abstract])) OR (“eMM”[Title/Abstract])) OR ("health information technology"[Title/Abstract])) OR (“reminder system*”[Title/Abstract])) OR (“Medical Informatics”[Title/Abstract]))

Nudge Terms
((((((((((((((((Choice Behaviour[MeSH Terms]) OR (Choice Behavior[MeSH Terms]) OR (Approach Behavior[MeSH Terms])) OR (Choice Architecture[MeSH Terms]) OR (“nudg*”[Title/Abstract])) OR (Behavioral Economics[MeSH Terms])) OR (“public commitment”[Title/Abstract])) OR (“social norm feedback”[Title/Abstract])) OR (“active choice”[Title/Abstract])) OR (“opt in"[Title/Abstract])) OR (“opt out"[Title/Abstract])) OR ("human behavio*"[Title/Abstract]))))))

Hospital Terms
((((((Emergency Department[MeSH Terms])) OR (Intensive Care Unit[MeSH Terms])) OR (“hospitalization”[Title/Abstract])) OR ("ICU"[Title/Abstract])) OR (“acute care”[Title/Abstract])) OR (“inpatient”[Title/Abstract]

Supplementary File 3: Assignment of overall risk of bias score

**Overall risk of bias**	**ROBINS-I tool descriptors(20)**	**Cochrane EPOC risk of bias tool descriptors(19)**
Low	The study was judged to be **low risk of bias for all domains**	The study was rated **'yes' for all domains**
Moderate	The study was judged to be at **low or moderate risk of bias for all domains**	The study was rated **'no' in a maximum of four domain**
Serious	The study was judged to be at **serious risk of bias** in at least one domain, but not at critical risk of bias in any domain	The study was rated **'no' in more than four domains**
Critical	The study was judged to be at **critical risk of bias in at least one domain**	The study was rated **'no' in all domains**
No information	There was no clear indication that the study is at serious or critical risk of bias *and* there was a lack of information in one or more key domains of bias	There was no clear indication of that the study is at serious risk of bias *and* one or more domains were rated as 'unclear'

ROBINS-I is Risk of Non-randomised Studies of Interventions, EPOC is Effective Practice and Organisation of Care Group.

Supplementary File 4: Characteristics of included studies examining effect of nudge interventions in electronic health records

Author, year	Country	Sample size (pre & post intervention)	Setting (ward/s, hospital/s, EHR)	Electronic health record	Study design	Pre/Post Data collection periods	Intervention target	Number of nudges implemented	Overall risk of bias
Astorga, 2019(23)	USA	Pre: 564; Post: 639 patients	NICU (39 bed); teaching hospital	Not reported	Interrupted time series	May 2013 - April 2014; May 2014 - May 2015	Medication/fluid use	1	Moderate
Bourdeau, 2014(40)	England	Intervention 1 pre:591, post: 1640; Intervention 2 pre: 2177, post:4022	ICU; teaching hospital (1200 admissions/year)	Draeger	Interrupted time series	Nov 2008 - Dec 2010; Feb 2010 - Nov 2012	Medication/fluid use	2	Serious
Coughlin, 2020(24)	USA	Pre: 87,084; Post: 206,466 patients	ED; 1 teaching (103000 admissions/year), 1 community hospital (60000 admissions/year), and 1 freestanding ED (25000 admissions/year)	EPIC Systems (Verona, WI)	Interrupted time series	Oct 2015 – Apr 2016; Apr 2016 – Jul 2017	Laboratory test orders	1	Moderate
Durand, 2013(25)	USA	34,776 orders (total)	All wards; 1025-bed teaching hospital	Not reported	Controlled before-after	Nov 2008 – May 2009; Nov 2009 – May 2010	Image ordering	1	Serious
Delgado, 2018(37)	USA	3264 prescriptions (total)	ED; 1 teaching, 1 community ‘medical centre’	EPIC Systems (Verona, WI)	Interrupted time series	Oct 2014 - March 2015; March 2015 - June 2015	Medication/fluid use	1	Serious

Feldman, 2013(26)	USA	Pre: 458,297; Post: 416,805 orders	All wards; 1051-bed 'tertiary care' hospital	Sunrise Clinical Manager (Allscripts Corp)	Controlled before-after	Nov 2008 – May 2009; Nov 2009 – May 2010	Laboratory test ordering	1	Moderate
Gerard, 2008(27); Trick, 2009(22)	USA	Year 1: 114; Year 2: 204; Year 3: 805 patients	Internal medicine ward; 464-bed public hospital	Cerner Inc. (Kansas City, MO)	Controlled before-after	Three three-month periods in 2004, 2005 and 2006	Medication/flui d use	2	Moderate
Herman, 2021(38)	Canada	Pre: 199; Post: 165 patients	All wards; 1400 inpatient beds distributed between 2 sites	Not reported	Interrupted time series	Jan 2016 – March 2017; March 2017 – June 2018	Medication/flui d use	1	Low
Iturrate, 2016(28)	USA	92,799 patients (total)	All wards; 930-bed paediatric teaching hospital	EPIC Systems (Verona, WI)	Before-after (no control)	June 2013 – June 2014; June 2014 – June 2015	Laboratory test ordering	1	No informatio n
Jacobs, 2012(29)	USA	Pre: 245; Post: 213 patients	All wards; 423-bed paediatric community hospital	Siemens Medical Solutions (Malvern, PA)	Before-after (no control)	Sep 2004 – Nov 2004; Dec 2005 – Feb 2006	Appropriate care	1	Serious
Leis, 2014(39)	Canada	Pre: 65 (28 catheterized); Post: 86 (49 catheterized) patients	All wards; 472-bed teaching hospital	Not reported	Controlled before-after	Jan 2013 and June 2013; Feb 2013 and July 2013	Medication/flui d use	1	No informatio n
Munigala, 2018(30)	USA	Pre: 3711; Post: 2788 patients	ED; teaching 'medical centre'	Not reported	Before-after (no control)	Sep 2015 – Oct 2015; Oct 2015 – Nov 2015	Laboratory test ordering	1	Serious

Muniga, 2020(31)	USA	Pre: 259; Post: 293 patients	ICU; hospital not reported	Not reported	Before-after (no control)	May 2018 – Aug 2018; Aug 2018 – Nov 2018	Medication/fluid use	3	Serious
Olson, 2015(32)	USA	7,578 prescriptions (total)	All wards; 550-bed teaching 'medical facility'	Cerner Connected Power chart (Cerner Corporation, Kansas City, MO, USA)	Before-after (no control)	Jan 2012 – April 2012; April 2012 – Jan 2013	Laboratory test ordering	2	No information
Rubins, 2019(33)	USA	Pre: 522; Post: 641 patients	All wards; 793-bed teaching 'medical center' & 162-bed teaching hospital	EPIC Systems (Verona, WI)	Interrupted time series with control	Jan 2017 – Sep 2017; Sep 2017 – May 2018	Appropriate care	1	No information
Sadowski, 2017(34)	USA	Intervention 1 pre: 2785, post: 2850; Intervention 2 pre: 2785, post: 3288 (total inpatient days)	General, cardiology, oncology wards; teaching 'tertiary care facility'	Not reported	Before-after (no control)	Jan 2014 – Feb 2014; Jan 2015 – Feb 2015; Jan 2016 – Feb 2016	Laboratory test ordering	2	No information
Sedrak, 2017(35)	USA	142,921 patients (total)	Not reported	Sunrise Clinical Manager (Allscript Corp)	Randomised controlled trial	April 2014 – April 2015; April 2015 – April 2016	Laboratory test ordering	1	Moderate

Smith, 2019(36)	USA	Pre: 283; Post: 286 caesarean deliveries	Caesarean delivery ward; 'Naval Medical Centre'	Not reported	Before-after (no control)	Aug 2017 – Nov 2017; Dec 2017 - March 2018	Medication/fluid use	1	Moderate

Supplementary File 5: Quality assessment for non-randomised studies using the ROBINS-I tool (Risk of Bias in Non-randomised Studies of Intervention)

**Author, year**	**Bias due to confounding [a]**	**Bias in selection of participants into the study [b]**	**Bias in classifications of interventions [c]**	**Bias due to deviations from intended interventions [d]**	**Bias due to missing data [e]**	**Bias in measurement of outcomes [f]**	**Bias in selection of the reported result [g]**	**Overall risk of bias score**
Astorga, 2019(23)	Low	Low	Moderate	Low	Moderate	Moderate	Low	Moderate
Bourdeaux, 2013(40)	Serious	No information	Low	Low	Moderate	Moderate	Low	Serious
Coughlin, 2020(24)	Low	Low	Low	Low	Moderate	Low	Low	Moderate
Delgado, 2018(37)	No information	Low	Low	Low	No information	Serious	Moderate	Serious
Durand, 2013(25)	Low	Low	Low	Low	Low	Serious	Low	Serious
Feldman, 2013(26)	Low	Low	Low	Low	Low	Moderate	Low	Moderate
Gerard, 2008(27)	Moderate	Low	Low	Moderate	Moderate	Low	Low	Moderate
Iturrate, 2016(28)	No information	Low	Low	Low	No information	Moderate	Low	No information
Jacobs, 2012(29)	Low	Low	Low	Low	Low	Serious	Low	Serious
Herman, 2021(38)	Low	Low	Low	Low	Low	Low	Low	Low
Leis, 2014(39)	Low	Low	Low	Low	Moderate	No information	Low	No information
Muniga, 2020(31)	Low	Low	Low	Low	Low	Serious	Low	Serious
Munigala, 2018(30)	Low	Low	Low	Low	Moderate	Serious	Low	Serious

Olson, 2015(32)	No information	Low	Low	Low	Low	Low	Low	No information
Rubins, 2019(33)	No information	Low	Low	Low	Low	Moderate	Low	No information
Sadowski, 2017(34)	No information	Low	Low	Low	Low	Moderate	Low	No information
Smith, 2019(36)	Low	Low	Low	Low	Low	Moderate	Low	Moderate
Trick, 2009(22)	Low	Low	Low	Serious	Low	Low	Low	Serious

[a]: Baseline confounding occurs when one or more prognostic variables (factors that predict the outcome of interest) also predicts the intervention received at baseline. ROBINS-I can also address time-varying confounding, which occurs when individuals switch between the interventions being compared and when post-baseline prognostic factors affect the intervention received after baseline

[b]: When exclusion of some eligible participants, or the initial follow-up time of some participants, or some outcome events is related to both intervention and outcome, there will be an association between interventions and outcome even if the effects of the interventions are identical. This form of selection bias is distinct from confounding—A specific example is bias due to the inclusion of prevalent users, rather than new users, of an intervention.

[c]: Bias introduced by either differential or non-differential misclassification of intervention status. Non-differential misclassification is unrelated to the outcome and will usually bias the estimated effect of intervention towards the null. Differential misclassification occurs when misclassification of intervention status is related to the outcome or the risk of the outcome and is likely to lead to bias.

[d]: Bias that arises when there are systematic differences between experimental intervention and comparator groups in the care provided, which represent a deviation from the intended intervention(s). Assessment of bias in this domain will depend on the type of effect of interest (either the effect of assignment to intervention or the effect of starting and adhering to intervention).

[e]: Bias that arises when later follow-up is missing for individuals initially included and followed (such as differential loss to follow-up that is affected by prognostic factors); bias due to exclusion of individuals with missing information about intervention status or other variables such as confounders.

[f]: Bias introduced by either differential or non-differential errors in measurement of outcome data. Such bias can arise when outcome assessors are aware of intervention status, if different methods are used to assess outcomes in different intervention groups, or if measurement errors are related to intervention status or effects.

[g]: Selective reporting of results in a way that depends on the findings and prevents the estimate from being included in a meta-analysis (or other synthesis).

Supplementary File 6: Quality assessment of randomised trials using the Cochrane Effective Practice and Organisation of Care Group (EPOC) methodology

	**Random component in the sequence generation process is described**	**Allocation concealment**	**Performance or patient outcomes were measured prior to the intervention, and no important differences were present across study groups**	**Baseline characteristics of the study and control providers are reported and similar**	**Missing outcome measures were unlikely to bias the results**	**Was knowledge of the allocated interventions adequately prevented during the study**	**Allocation was by community, institution or practice and it is unlikely that the control group received the intervention**	**There is no evidence that outcomes were selectively reported**	**Was the study free from other risks of bias**	**Overall risk of bias score**
Sedrak, 2017(35)	Y	Y	Y	Y	Y	N*	Y	Y	Y	Moderate

* Owing to the nature of the intervention, clinicians could not be blinded to group assignment. All investigators, statisticians, and data analysts were blinded to the results until the study was completed.

Supplementary File 7: Outcomes of included studies of nudge interventions in electronic health records and risk of bias

Author, year	Primary outcome measure(s)	Results following intervention	Post-intervention time frame	Risk of bias
**Decision structure – change choice defaults**				
Astorga, 2019(23)	Rate of parenteral antibiotic use per patient-day	Improvement: Rate reduction of 25% (p < .0001)	Monthly interrupted time-series analysis showed a steady reduction over one year (95% confidence interval: –10.42 to –2.23)	Moderate
Bourdeaux, 2014*(40)	Intervention 1: Percentage of prescriptions of chlorhexidine per patient per month.	Improvement: Significant increase from 55.3% to 90.4%	Monthly interrupted time-series analysis showed a steady reduction over 3.5 years	Serious
Delgado, 2018(37)	Percentage of prescriptions written per default option of 10 or 20 tablets.<br>Mean number of tablets prescribed (oxycodone 5mg/acetaminophen 325 mg)	Improvement: The proportion of prescriptions written for 10 tablets increased from 20.6% to 43.3% (difference of 22.8%, 95% CI: 19.6, 25.9%)<br>Improvement: The proportion for prescriptions written for 20 tablets decreased from 22.8% to 16.1% (difference of -6.7%, 95% CI: -9.4%, -4%)<br>No change: No change in mean number of tablets prescribed (p=0.42)	Weekly interrupted time-series analysis showed an increase over 18 weeks (95% confidence interval: 19.6 to 25.9)<br><br>Weekly interrupted time-series analysis showed a decrease over 18 weeks (95% confidence interval: -9.4 to -4.0)	Serious
Jacobs, 2012*(29)	Percentage of patients with an order for: admission weight, activity centre visits, and peak flow	Improvement: The percentage of patients with order for admission weight (79.2% to 94.8%, p<0.001), activity centre (84.1% to 95.3%, p<0.001) and peak flow (18.8% to 55.9%, p<0.001)	3-month data collection period	Serious
Leis, 2014(39)	Change in treatment of asymptomatic bacteriuria (ASB) in non-catheterized inpatients	Improvement: There was an absolute risk reduction of 36% (95% CI, 15%–57%) in ASB treatment	6-month data collection period	No information

Olson, 2015*(32)	Intervention 1: Percentage of transfusions with hematocrit count orders<br>Intervention 2: Percentage of transfusions with platelet count orders	1) Improvement: Increase from 8.3% to 57.5% (P<0.0001)<br><br>2) Improvement: Increase from 7.0% to 59.4% (P<0.001)	13-week data collection period	Moderate
Rubins, 2019(33)	Percentage of patients with telemetry orders	Improvement: Reduction in telemetry ordering from 79.1% of patients to 21.3% (P<0.001)	Reduction consistent over 7.5 month follow-up	No information
Gerard, 2008; Trick, 2009*	Percentage of patients vaccinated for influenza	Improvement: 36% of patients (compared to <5% for controls in previous year, p<0.001)^	3-month data collection period	Moderate
**Decision information – make information visible**				
Coughlin, 2020(24)	Number of urine culture orders per 100 patients	Improvement: decrease of 2.98 urine cultures per 100 orders (95% CI: 1.45, 4.51)	Reduction sustained for a 1-year follow-up period	Moderate
Durand, 2013(25)	a) Mean relative utilization change for laboratory test ordering<br>b) Total charges associated with the test orders	a) No change b) No change	6-month data collection period	Serious
Feldman, 2013(26)	a) Total number of orders placed<br>b) Rate of ordered tests per patient-day<br>c) Total charges associated with the test orders	a) Improvement: Reduction in tests ordered by 9.1% (p<0.001)<br>b) Improvement: 3.72 tests per patient-day to 3.40 (8.59% decrease; 95% confidence interval: -8.99% to -8.19%)<br>c) Improvement: Net charge reduction of $436,115 (all P<0.001)	6-month data collection period	Moderate
Herman, 2021(38)	a) Rate of antibiotic orders (metronidazole, oral	a) Improvement: Decrease from 13.6 (10-16) to 7.9 days (1-13) - (difference −5.8	Monthly interrupted time-series analysis showed the change was generally	Low

	vancomycin, and fidaxomicin) as days of therapy per patient<br>b) Proportion of patients receiving no antibiotic therapy	days; 95% confidence interval, −3.9 to −7.6)<br>b) Improvement: Increase from 6.5% to 23.6% (OR, 4.5; 95% CI, 2.3–8.7)<br>(both P<0.0001)	maintained over one year though with some variation and at times no change was observed	
Sadowski, 2017*(34)	Percentage change in the number routine tests ordered per patient day	Improvement: Reduction of 15.3% (P<0.001)	2-month data collection period	No information
Sedrak, 2017(35)	Number of tests ordered per patient-day	No significant overall change in outcomes (all P>0.05)<br>A significant relative decrease in test ordering for patients with an ICU stay (−0.16 tests ordered per patient-day; 95% CI, −0.31 to −0.01; P = .04) was offset by a significant relative increase in test ordering for patients without an ICU stay (0.13 tests ordered per patient-day; 95% CI, 0.08-0.17; P < .001)	Data collection period of one year	Moderate
**Decision structure – change range or composition of options**				
Muniga, 2020*(31)	Intervention 1) Percentage of patients with orders for scheduled acetaminophen<br><br>Intervention 2) Percentage of patients with orders for H2RA	Intervention 1) No change: no orders made pre or post<br><br>Intervention 2) Improvement: Increase in the percentage of patients prescribed an H2RA for stress ulcer prophylaxis (0 vs. 20%, p<0.001)	3-month data collection period	Serious
Minugala, 2018(30)	Daily culture rate per 1000 patients (urine testing)	Improvement: Rate decreased by 46.6% (95% CI −66.2% to –15.6%), although urinalysis, microscopy, and catheterised urine culture rates did not change .	41-day data collection period	Serious

Sadowski, 2017* (34)	Number routine tests ordered per patient day	Improvement: Decrease from 4.99 to 4.02 (p <.001)	2-month data collection period	No information
Smith, 2019(36)	a) Median morphine milligram equivalents per hospital stay<br>b) The median ibuprofen dose per day per patient<br>c) Percentage of patients receiving ketorolac<br>d) Acetaminophen use per day<br>e) Percentage of patients with post-caesarean patient-controlled analgesia use	a) Improvement: reduction of 75% (from 120 (90–176 IQR) to 30 (5–68) postintervention (P< .001)<br>b) No change<br>c) No change<br>d) Improvement: increase in acetaminophen use per day (from 753 mg to 2,340 (P<.001)<br>e) No change	4-month data collection period	Moderate
**Decision assistance – provide reminders (make information more or less salient)**				
Muniga, 2020*(31)	Percentage of patients with order for lactated Ringer's solution	No change: Reduction from 17% to 4% (p=0.005); however normal saline orders also decreased over the same period (18% vs 9%, p=0.06) due to a lower proportion of patients requiring IV fluids.	3-month data collection period	Serious
Gerard, 2008(21); Trick, 2009* (22)	Percentage of patients vaccinated for influenza	No change: 6.0% vaccinated compared to 1% in control group.	3-month data collection period	Serious
**Decision structure – change option-related effort**				
Iturrate, 2016(28)	a) Total number of laboratory test orders<br>b) Total charges associated with the test orders	a) Improvement: reduction of 8.52% in targeted laboratory tests (p<0.001)<br>a) Improvement: Estimated savings of $323,489	Segmented regression analysis showed a small but significant upward trend (slope of 0.0004, P<.001) suggesting an overall return to baseline rates. The two most ordered tests returned to baseline rates within one year.	No information
Bourdeaux, 2014*(40)	Percentage of patients receiving HEC per month.	Improvement: decrease from 54.1% to 3.1% (p<0.001)	Monthly interrupted time-series analysis showed a	Serious

			steady reduction over 3.5 years.	

HEC is hydroxyethyl starch, ICU is intensive care unit, ASB is asymptomatic bacteria
*These studies used multiple nudge interventions
^These results are for the two interventions implemented simultaneously i.e pre-selected vaccine order for doctors and reminder for nurses
